# Targeting Oxidative Stress in Septic Acute Kidney Injury: From Theory to Practice

**DOI:** 10.3390/jcm10173798

**Published:** 2021-08-25

**Authors:** Connie P. C. Ow, Anton Trask-Marino, Ashenafi H. Betrie, Roger G. Evans, Clive N. May, Yugeesh R. Lankadeva

**Affiliations:** 1 Preclinical Critical Care Unit, Florey Institute of Neuroscience and Mental Health, Melbourne, VIC 3052, Australia; ow.peichenconnie73@ncvc.go.jp (C.P.C.O.); traskmarinoanton@gmail.com (A.T.-M.); ashenafi.betrie@florey.edu.au (A.H.B.); roger.evans@monash.edu (R.G.E.); clive.may@unimelb.edu.au (C.N.M.); 2 Department of Cardiac Physiology, National Cerebral and Cardiovascular Center Research Institute, Osaka 564-8565, Japan; 3 Melbourne Dementia Research Centre, Florey Institute of Neuroscience and Mental Health, Melbourne, VIC 3052, Australia; 4 Cardiovascular Disease Program, Biomedicine Discovery Institute and Department of Physiology, Monash University, Melbourne, VIC 3800, Australia; 5 Department of Critical Care, Melbourne Medical School, University of Melbourne, Melbourne, VIC 3052, Australia

**Keywords:** septic acute kidney injury, oxidative stress, hypoxia, N-acetylcysteine, vitamin C

## Abstract

Sepsis is the leading cause of acute kidney injury (AKI) and leads to increased morbidity and mortality in intensive care units. Current treatments for septic AKI are largely supportive and are not targeted towards its pathophysiology. Sepsis is commonly characterized by systemic inflammation and increased production of reactive oxygen species (ROS), particularly superoxide. Concomitantly released nitric oxide (NO) then reacts with superoxide, leading to the formation of reactive nitrogen species (RNS), predominantly peroxynitrite. Sepsis-induced ROS and RNS can reduce the bioavailability of NO, mediating renal microcirculatory abnormalities, localized tissue hypoxia and mitochondrial dysfunction, thereby initiating a propagating cycle of cellular injury culminating in AKI. In this review, we discuss the various sources of ROS during sepsis and their pathophysiological interactions with the immune system, microcirculation and mitochondria that can lead to the development of AKI. We also discuss the therapeutic utility of N-acetylcysteine and potential reasons for its efficacy in animal models of sepsis, and its inefficacy in ameliorating oxidative stress-induced organ dysfunction in human sepsis. Finally, we review the pre-clinical studies examining the antioxidant and pleiotropic actions of vitamin C that may be of benefit for mitigating septic AKI, including future implications for clinical sepsis.

## 1. Introduction

Sepsis is the leading cause of acute kidney injury (AKI), accounting for approximately 50% of cases of renal dysfunction in intensive care units [1]. Development of AKI during sepsis is both a significant and an independent prognostic factor for prolonged hospitalization and in-hospital death [2]. There is also mounting epidemiological evidence that survivors of either mild or short episodes of AKI are predisposed to greater risk of developing chronic kidney disease (CKD) and end-stage renal disease in later life [3]. Antibiotics and resuscitation with fluids and vasopressors are currently the mandated treatments for human sepsis. Renal replacement therapy (RRT) is recommended for patients who develop severe septic AKI [4]. However, these are mainly palliative interventions aimed at keeping the patients alive in the hope that the kidneys recover. Accordingly, a better understanding of the pathophysiology of septic AKI is required to formulate effective mechanism-guided interventional strategies.

Although global renal ischemia has been proposed as a cause of septic AKI [5], there is both experimental and clinical evidence challenging this dogma. Histopathological investigations performed on post-mortem kidney tissue from patients that succumbed to septic AKI demonstrate heterogenous focal, patchy tubular injury, with minimal tubule-epithelial death (<5%), apical vacuolization and minor focal mesangial expansion, which are not characteristic of severe renal ischemic injury [6,7]. In addition, there is compelling evidence from clinically relevant ovine and porcine models of sepsis that AKI develops even in the absence of global renal ischemia [8,9,10]. Similarly to the histopathological findings in human sepsis, acute tubular necrosis and tubular cell apoptosis were not characteristic of AKI in such large mammalian models of hyperdynamic sepsis [9,11]. During inflammatory conditions such as sepsis, there appears to be an uncoupling of the renal microcirculation from the macrocirculation [12]. In ovine sepsis, selective renal medullary tissue ischemia and hypoxia precede the development of AKI by 12–24 h, despite increases in renal blood flow and renal cortical perfusion and oxygenation [13,14,15]. Oxidative stress plays a critical role in promoting adaptive responses to localized tissue hypoxia by stabilization of hypoxia inducible factors (HIF), which promotes the transcription of multiple genes [16]. However, in sepsis there is an imbalance between reactive oxygen species (ROS) and reactive nitrogen species (RNS) and the host’s antioxidant defense mechanisms. In this review, we outline the sources and roles of oxidative and nitrosative stress in the pathophysiology of septic AKI with an emphasis on its interactions with inflammation, microcirculatory abnormalities, tissue hypoxia and mitochondrial dysfunction. We also outline recent pre-clinical and clinical studies that have investigated the use of antioxidants, principally N-acetylcysteine (NAC) and vitamin C, as a potential therapeutic strategy for septic AKI.

## 2. Interactions between the Septic Inflammatory Cascade and Oxidative Stress

**Figure 1:**
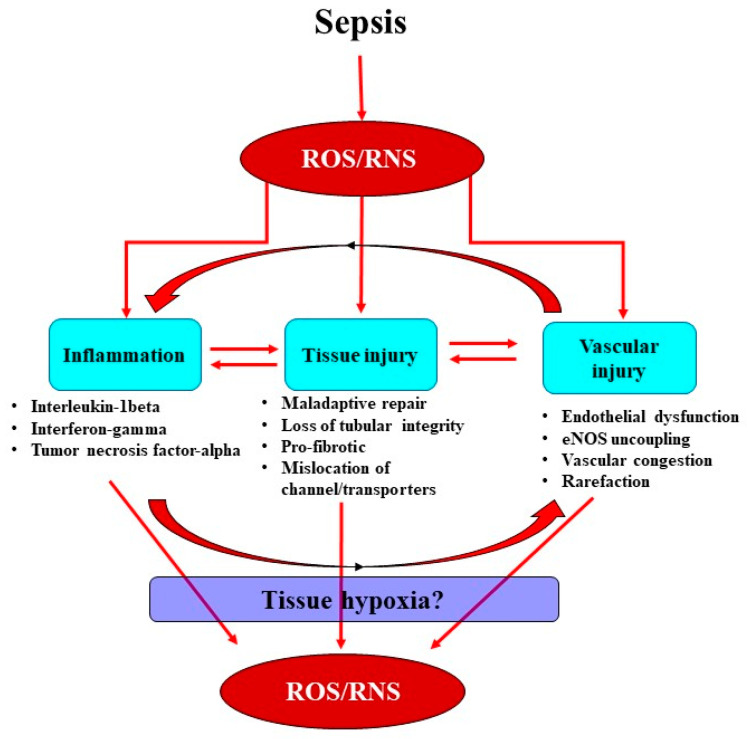
Schematic of the proposed relationships among sepsis-induced production of reactive oxygen species (ROS) and reactive nitrogen species (RNS) and inflammation, tissue, and vascular injury. ROS and RNS result in enhanced production of immune-modulatory cells at sites of injury in vessels and tubules, thereby initiating a complex cascade of inflammation and injury. Importantly, tissue and vascular injuries in severe sepsis can have deleterious consequences, as they can contribute to an uncoupling of endothelial nitric oxide synthase (eNOS), mediating endothelial dysfunction and tissue hypoxia, thereby enhancing the accumulation of ROS and RNS.

The inflammatory response is the body’s first line of defense against invading pathogens, but this can also be a critical initiating factor for renal injury. In sepsis, inflammatory mediators, including pathogen- and damage-associated molecular patterns, are released into the intravascular area and are detected by Toll-like receptors on tubular and endothelial cells [17]. Activation of these receptors subsequently propagates a myriad of downstream processes contributing to tubular reparation, vascular rarefaction and amplification of pro-inflammatory immune modulators at sites of injury, leading to vascular congestion and endothelial dysfunction [18]. These processes appear to converge to stimulate superoxide-induced amplification of tissue hypoxia and cellular injury (Figure 1).

**Figure 2:**
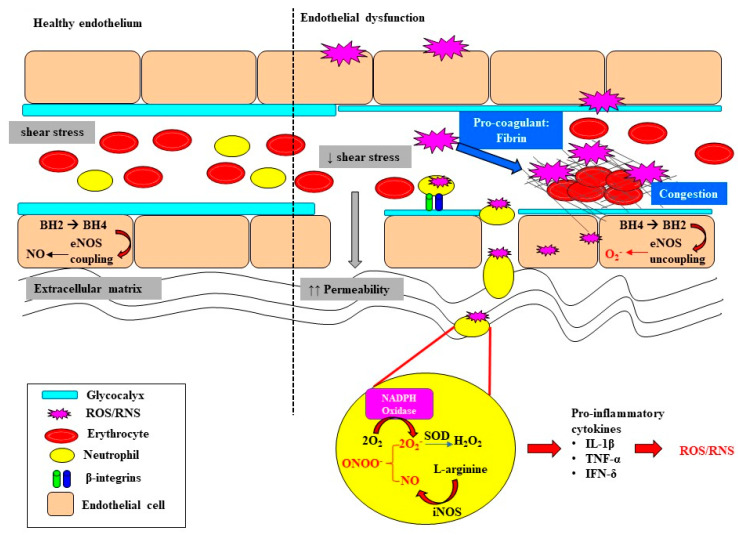
A schematic outlining the initiation of oxidative damage to the endothelium, the downstream consequences for endothelial dysfunction and the propagation of oxidative stress. The layer of glycocalyx lining the apical surface of endothelial cells is important for the maintenance of shear-stress and flow-mediated nitric oxide (NO) release and vasodilation of the endothelium. Sepsis-induced oxidative stress leads to glycocalyx thinning and shedding, resulting in the loss of gap junctions, and subsequently increased permeability and vascular leakage. Loss of gap junctions also greatly facilitates extravasation of neutrophils in the circulation, mediated by β-integrins, through to the extracellular matrix. The resultant superoxide (O_2_^−^) that is formed can interact with nitrosative species generated by inducible nitric oxide synthase (iNOS), forming the highly reactive peroxynitrite (ONOO^−^) and then hydrogen peroxide (H_2_O_2_). Oxidative bursts from neutrophils contribute to enhanced oxidative damage and the downstream production of pro-inflammatory cytokines which have the intrinsic capability of producing reactive oxygen species (ROS) and reactive nitrogen species (RNS). Oxidative stress can also induce the recruitment of pro-coagulants to the site of injury, resulting in vascular congestion, ultimately impeding blood flow. Increased oxidative stress at the endothelium depletes and oxidizes the pool of tetrahydrobiopterin (BH4), resulting in the uncoupling of endothelial nitric oxide synthase (eNOS) in endothelial cells, thereby enhancing the production of ROS. Interleukin-1beta (IL-β); tumor necrosis factor-alpha (TNF-α), interferon-gamma (IFN-δ).

Sepsis-induced tubular and vascular injuries, in concert with oxidative stress, trigger the recruitment of polymorphonuclear neutrophils, setting in motion a cascade of immunomodulatory events which leads to downstream production of ROS and RNS, further propagating injury [19]. Neutrophils have the ability to generate superoxide through a complex process known as the “oxidative burst” [20,21]. Neutrophils express multiple receptors, including the β1, β2 and β3 integrins, which when activated bind to fibronectin, fibrinogen and collagen, mediating their translocation to the extracellular matrix by P-selectins and E-selectins [22]. Here, a series of signaling cascades ultimately leads to the downstream release of intracellular calcium and the formation of the nicotinamide adenine dinucleotide phosphate (NADPH) oxidase complex, resulting in the production of ROS [20,21]. Furthermore, inducible nitric oxide synthase (iNOS) activity is upregulated within immune cells (Figure 2). This increases the production of nitric oxide (NO), which can react with the ROS produced by NADPH oxidase to form the RNS peroxynitrite (Figure 2). Peroxynitrite contributes to nitrosative damage, such as S-nitrosylation of proteins, thereby affecting normal functioning of proteins. The notion of ROS-induced vascular injury is supported by the observation in patients with sepsis of extensive production of superoxide from leucocyte microparticles, which in turn enhances adhesion molecules’ activity and endothelial activation [23]. Moreover, high plasma levels of lipid peroxidation markers (F2-isoprostanes and isofurans) have been strongly associated with AKI in patients with sepsis [24].

The generation of oxidative stimuli by neutrophils further attracts the pro-inflammatory chemokine ligand-5 and intracellular adhesion molecule-1, which are important factors that facilitate the recruitment of leukocytes to sites of tissue injury [25,26]. Mobilized and activated leukocytes initiate a cytokine storm involving the recruitment of pro-inflammatory cytokines, including interleukin-1-beta (IL-1β), interferon-gamma (IFN-γ) and tumor necrosis factor-alpha (TNF-α) [27,28], which can lead to the further production of ROS (Figure 1). Indeed, inflammatory and oxidative stress biomarkers such as TNF-α, IL-1β, myeloperoxidase activity, malondialdehyde and hydrogen peroxide (H_2_O_2_) are all reported to be significantly elevated in patients with septic AKI [29]. Collectively, the initial inflammatory cascade in sepsis appears to be a critical initiating factor in the propagation of oxidative stress, which can have deleterious effects on renal microcirculation.

## 3. Oxidative Stress Exacerbates Microcirculatory Abnormalities and Vascular Rarefaction

Endothelial dysfunction and microvascular rarefaction have been described as common pathophysiological features of AKI and are postulated to be critical factors mediating progression to CKD following recovery from AKI [30,31,32]. The NO system is an important regulator of vascular tone within the renal microcirculation, but it can be deleteriously affected in sepsis (Figure 2). In the healthy state, the biosynthesis of NO by vascular endothelial cells is dependent on the coupling state of endothelial nitric oxide synthase (eNOS) and the bioavailability of the co-factor tetrahydrobiopterin (BH4) [33]. When endogenous levels of the co-factor BH4 are sufficient, L-arginine is coupled with the reduction of oxygen, leading to the production of the potent vasodilator NO [33,34]. However, when BH4 levels are low, eNOS is uncoupled and superoxide is produced instead. Furthermore, BH4 is highly susceptible to oxidization to BH2 when levels of superoxide are high, further depleting the pool of the rate limiting co-factor BH4 [35,36]. Uncoupling of eNOS is reported to contribute to the pathophysiology of a myriad of kidney diseases arising from diabetes, hypertension and ischemia-reperfusion injury [37,38]. Intravenous supplementation with BH4 in an ovine model of sepsis improved microvascular dysfunction via increasing the number of perfused vessels, the proportion of perfused small vessels and the microvascular index within the sublingual circulation [39]. In an ovine model of severe septic AKI induced by intravenous infusion of live *Escherichia Coli* for 48 h, eNOS gene expression was selectively down regulated in the renal medulla, but not the renal cortex [11]. However, whether an uncoupling of eNOS contributes to the early onset of microcirculatory abnormalities reported within the renal medulla in ovine septic AKI [13] warrants further investigation.

In sepsis, excessive superoxide generation and accumulation, in tandem with inflammation, also results in direct structural damage to the vasculature, resulting in vascular leakage and tissue edema (Figure 2) [40]. The endothelial glycocalyx is a crucial regulator of endothelial function and vascular tone. The endothelial glycocalyx lining the apical surface of the endothelium, consisting of hyaluronic acid, heparan sulphate, glycoproteins and proteoglycans, ensures the integrity of vascular permeability, so it protects against vascular leakage, coagulation and persistent inflammation (Figure 2) [41,42]. It is also an important mechano-transducer, as it senses shear stress, thereby contributing to shear-stress mediated NO production and vasodilation. Under pathological conditions characterized by oxidative stress, the production of hyaluronidases that catalyze the depolymerization and degradation of hyaluronic acid of the glycocalyx is upregulated. The resultant shedding and thinning of the endothelial glycocalyx not only increases vascular permeability [41,43], but also compromises microcirculatory perfusion in sepsis [44,45]. For example, the thinning and shedding of the endothelial glycocalyx can decrease the ability of the endothelium to respond to shear-stress-mediated release of NO (Figure 2) [45]. Plasma levels of endothelial glycosaminoglycans in patients who survived sepsis were double those compared with healthy volunteers [46]. Moreover, the shedding of vascular endothelial cadherin, a protein essential for maintenance of gap junctions between endothelial cells, was significantly greater in septic patients with AKI that required RRT compared with those without AKI [47]. These clinical observations support the notion that the loss of endothelial integrity may play a crucial role in driving microcirculatory abnormalities in septic AKI.

The damaged endothelium also attracts leukocytes to the site of injury, as part of the innate immune response facilitated by the exposed intercellular and vascular cell adhesion molecules. This homing of pro-inflammatory cells, in conjunction with compromised gap junctions, leads to extravasation of the pro-inflammatory cells from the endothelium into the surrounding tissue, contributing to persistent inflammation [48]. Notably, inflammatory cells can generate ROS themselves and so reduce NO bioavailability [48], thereby contributing to the extensive pool of superoxide, essentially setting up a vicious cycle of oxidative stress, inflammation and vascular injury [19] (Figure 1). Sepsis-induced microvascular injury can also release microparticles into the systemic circulation. Microparticles are cell membrane-derived particles that consist of vasoconstrictive compounds such as prostaglandin E2, thromboxane A2, inflammatory mediators, pro-coagulants and fibrin. Thus, microparticles can act as localized sources of oxidative stress, inflammation and disseminated intravascular coagulopathy, resulting in vascular congestion [49]. It is conceivable that these microcirculatory perturbations may be amplified, especially within the smaller arteries of the renal medullary vasa recta, which may explain why the renal medulla is susceptible to developing hypoxia during sepsis.

## 4. Renal Medullary Tissue Hypoxia: A Critical Event in Acute Kidney Injury?

Renal medullary hypoxia is emerging as a common pathophysiological feature of AKI arising from sepsis [13,50], cardiopulmonary bypass [51,52] and radiocontrast-induced nephropathy [53]. Furthermore, renal medullary hypoxia has been implicated as an important driver in the transition and/or propensity for progression from AKI to CKD [54,55]. The relatively high metabolic requirements of the tubular elements in the renal medulla, coupled with the topography of vascular and tubular architecture within the medulla, result in a steep oxygen gradient between the capillaries (vasa recta) and both the thick and thin ascending limbs of the loop of Henle and the collecting ducts [56]. There is also the potential for diffusive oxygen shunting in the renal medullary microcirculation (from descending to ascending vasa recta), which could further compromise renal medullary oxygen delivery [57]. In healthy sheep, graded occlusion of the renal artery and thus progressive reductions in renal blood flow resulted in proportionally greater degrees of renal medullary ischemia and hypoxia compared with a renal cortex indicative of an intrinsic deficit in the autoregulatory capacity of the renal medullary microcirculation [58]. Accordingly, under pathophysiological settings such as sepsis, renal medullary microcirculatory perturbations leading to even modest reductions in medullary oxygen delivery or increases in oxygen consumption can have adverse consequences for medullary tissue oxygenation.

Renal medullary hypoxia can be a major driver of a cascade of events leading to cellular injury, vascular injury and tubular dysfunction [59]. Acute renal insults, including endotoxemia, can both increase renal tissue oxygen consumption and reduce tissue oxygen delivery. For example, these changes can result in tubular injury and obstruction, and mislocalization of Na/K-ATPase and transport proteins within renal tubular epithelial cells, thereby reducing the efficiency of oxygen utilization for sodium reabsorption [60]. In the context of the current review, it is also relevant that oxidative stress can increase tubular oxygen consumption both by reducing the efficiency of ATP production within the mitochondria and/or by enhancing oxygen utilization for tubular sodium reabsorption [61]. Moreover, renal insults that acutely compromise vascular integrity or chronically promote vascular rarefaction can reduce renal medullary oxygen delivery and further worsen tissue hypoxia. In a clinically relevant ovine model of gram-negative sepsis, the development of renal medullary hypoxia preceded AKI, as detected by elevated plasma creatinine and oliguria, by up to 12–24 h [13,14,15]. In the same model of ovine sepsis, bladder urinary oxygenation was validated to provide a reliable estimate of renal medullary tissue oxygenation during development of septic AKI [15] and in response to clinical interventions, including resuscitation with fluids [62], vasopressors [14] and diuretics [63]. In subsequent human studies, the presence of renal tissue medullary hypoxia, as indirectly assessed by measurement of bladder urinary hypoxia was reported in patients with septic AKI [64]. Cellular protective mechanisms driven by HIFs can protect the kidneys when tissue hypoxia is mild and/or brief, but they can fail when hypoxia is severe and/or protracted, as can occur during sepsis [65].

HIFs are cellular oxygen sensors. Stabilization of the α-subunit at low oxygen concentrations results in the formation of a dimer with the β-subunit. This dimer then translocates to the nucleus and binds to hypoxia response elements, resulting in altered transcription of a myriad of proteins essential for many key signaling pathways [66,67]. The upregulation and stabilization of HIFs is adaptive because it results in the production of erythropoietin, which promotes the production of red blood cells [68,69]. This increases blood oxygen carrying capacity, so it has the potential to ameliorate tissue hypoxia. Another important protein that is upregulated by the stabilization of HIFs is eNOS, which potentially increases NO bioavailability and ameliorates vasoconstriction, thereby limiting the further propagation of both hypoxia and ROS/RNS [66]. However, HIFs can be a double-edged sword because extensive production of HIFs in response to prolonged hypoxia in severe sepsis can result in excessive production of vasoconstrictive and ROS-inducing proteins, such as iNOS [70], and proteins that contribute to fibrogenesis [71]. Germane to such observations, an early onset of renal medullary hypoxia in sepsis resulting in prolonged phases of tissue hypoxia may lead to the destabilization of HIFs that propagate oxidative and nitrosative injuries, culminating in AKI. Supporting this notion, HIF-1α gene expression was selectively downregulated within the renal medulla in septic sheep with severe AKI after 48 h of sepsis [11].

It must be acknowledged that the mechanistic link between renal medullary hypoxia and reduced glomerular filtration rate remains unidentified in AKI and may differ depending on the etiology of AKI. It is, however, conceivable that prolonged tissue hypoxia, particularly in metabolically active regions of the kidney such as the renal medulla, induces mitochondrial dysfunction, resulting in increased ROS and thus further amplification of renal cellular injuries.

## 5. Sepsis-Induced Mitochondrial Dysfunction Activates Production of ROS

Mitochondrial dysfunction is proposed to be both a cause and consequence of renal hypoxia in the pathogenesis of septic AKI [72,73]. Mitochondria are the main consumers of oxygen within the kidneys. Thus, the production of physiological levels of mitochondrial ROS in the mitochondrial matrix is important because ROS serve as signals and regulators for a myriad of biological processes. These include adaptation to hypoxia through regulating the stabilization of HIFs [74]; facilitating the formation of autophagosomes through oxidation of the cysteine protease autophagy-related gene-4 by H_2_O_2_ [75]; and ROS-dependent activation of phosphoinositide-3 kinase, leading to downstream production of pro-inflammatory cytokines, including caspase-1, IL-1β and IL-18 [76]. However, as cells experience prolonged periods of hypoxia, there is a change in metabolism and poor utilization of the available oxygen for ATP production in the mitochondrial electron transport chain, resulting in increased leakage of electrons and elevated production of free radicals/ROS [77,78]. Mitochondria use oxygen as the final acceptor of the respiratory chain, but its incomplete reduction can also produce ROS, especially superoxide [79]. Complex III of the electron transport chain is the inherent oxygen sensor during acute hypoxia, and it regulates the production of superoxide inversely with oxygen availability [80]. The transition of complex I from the active to “de-active” form was also reported to have the capacity to produce ROS outbursts during acute hypoxia [81]. Patients with septic AKI have elevated levels of receptor-interacting protein kinase-3 (RIPK3) in urine and plasma [82]. RIPK3 promotes oxidative stress and mitochondrial dysfunction in kidney tubular epithelial cells by increasing the expression and mitochondrial translocation of NADPH oxidase 4 and inhibition of mitochondrial complexes I and III [82,83,84]. It is therefore not surprising that mitochondrial injury has been commonly related to multi-organ dysfunction in patients with sepsis [6,85].

**Figure 3:**
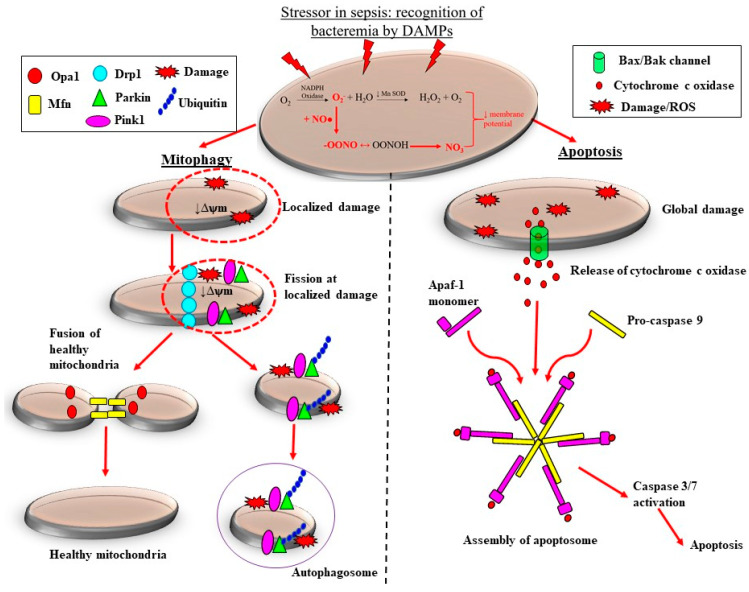
Adaptive and maladaptive responses of the mitochondria to sepsis-induced oxidative stress. Increased production of superoxide (O_2_^−^) by nicotinamide adenine dinucleotide phosphate (NADPH) oxidase coupled with the reduced activity of manganese superoxide dismutase (MnSOD) results in the accumulation of O_2_^−^. Cytosolic nitric oxide (NO) produced by inducible nitric oxide synthase (iNOS) reacts with O_2_^−^, forming the highly reactive peroxynitrite (ONOO^−^). The accumulation of O_2_^−^ and ONOO^−^ results in persistent oxidative stress and a reduction in mitochondrial membrane potential (ψm) and so mitochondrial dysfunction. In the case of localized mitochondrial damage, mitochondrial quality control mechanisms are activated which arrest mitochondrial dysfunction and limit excessive mitochondrial loss. Recruitment of mitochondrial fission proteins to sites of injury targets the damaged portions of the mitochondrion for fission. Subsequently, ubiquitin, PTEN-induced kinase (PINK1) and E3 ubiquitin-protein ligase (Parkin) proteins are recruited to the damaged mitochondrion, removed by mitochondrial fission and interact with phagophore, consequently forming an autophagosome. The healthy portion of the mitochondrion undergoes mitochondrial fusion, adding to the existing mitochondrial pool and limiting excessive mitochondrial loss. On the other hand, extensive damage to mitochondria in severe sepsis results in the release of cytochrome c oxidase, and in the formation of an apoptosome when it interacts with apoptotic protease activating factor-1 (Apaf-1) monomers and pro-caspase 9. This leads to downstream activation of caspase 3/7, ultimately resulting in the containment of sepsis-induced damage via apoptosis. Mitochondrial dynamin-like GTPase (OPA-1); dynamin related protein-1 (DRP-1); damage-associated molecular patterns (DAMPs).

There are pre-clinical and clinical studies demonstrating that the adaptive processes of mitochondrial fission are downregulated in sepsis, which likely contributes to the loss of mitochondrial mass, thereby propagating ROS-induced damage during septic AKI. Sepsis is associated with considerable morphological changes in mitochondria. These changes include reduced numbers of cristae due to swelling of the inter-cristae space and the mitochondrial matrix, and vacuolation within the mitochondria space [82,86]. Depending on the severity of mitochondrial damage, the removal of mitochondria can be carried out by two pathways: mitophagy and apoptosis (Figure 3). Localized and extensive morphological aberrations within the mitochondria can lead to the accumulation of superoxide, eventually causing the opening of mitochondrial membrane channels. This leads to reduced mitochondrial membrane potential, localized to the site of injury, thereby further enhancing production of ROS [87]. Furthermore, increasing levels and/or accumulation of ROS can result in the upregulation of uncoupling protein-1 [88], consequently resulting in excessive proton leakage and hampering the production of ATP [89]. In this case, because the damage tends to be localized, the mitochondrion is targeted for mitochondrial fission, followed by subsequent removal of the damaged portion of the mitochondrion by mitophagy (Figure 3). During mitochondrial fission, “pinching” of the mitochondrial membrane occurs at the injury site, which is facilitated by the assembly of dynamin-related protein-1 that facilitates the removal of the damaged portion of the mitochondria from the undamaged portion (Figure 3) [90]. Following this, PTEN-induced kinase 1 and E3 ubiquitin-protein ligase (Parkin) proteins are recruited to the damaged mitochondrion, which ultimately forms an autophagosome and is removed by mitophagy [90]. Mitochondrial fusion proteins, mitofusin and mitochondrial dynamin-like GTPase, are recruited to the remaining healthy portion of the mitochondrion, which then fuses with an adjacent healthy mitochondrion (Figure 3). Thus, mitochondrial fission, followed by fusion, in this case, is seemingly an important adaptive reparative process because it not only arrests mitochondrial damage, but also prevents excessive loss of mitochondrial mass, thereby limiting ROS-induced injury. Supporting this notion, decreased expression of both mitochondrial PINK1 and Parkin mRNA has been reported in biopsies derived from patients who succumbed to severe septic AKI [91].

Extensive damage to the mitochondria results in global reduction of mitochondrial permeability, which can lead to accumulation of superoxide eventually, causing the opening of mitochondrial membrane channels. These injuries trigger a series of downstream events, starting with the release of the pro-apoptotic factors Bcl-2-associated X protein BAX and B-cell lymphoma-2 protein. This mediates the release of cytochrome c, which in turn interacts with apoptotic protease activating factor-1 proteins, ultimately forming an apoptosome that triggers a procaspase-9-mediated downstream intrinsic apoptosis cascade [92,93] (Figure 3). This process facilitates the removal of whole mitochondria in severe sepsis and results in a reduction of mitochondrial mass [91], which can further compromise the production of host antioxidants and enhance the production of superoxide (Figure 3). Therefore, ROS-induced damage to the mitochondria in sepsis can result in enhanced ROS production and accumulation, which can contribute to the vicious propagating cycle of oxidative stress, microvascular injury and cellular injury, culminating in AKI. Theoretically then, development of pharmacological therapies aimed towards arresting and/or limiting the production and accumulation of ROS and/or RNS during sepsis should mitigate the propagation of vascular, mitochondrial and cellular injuries, and limit the severity of septic AKI. Below, we discuss pre-clinical and clinical studies that have investigated the use of NAC or vitamin C as a therapeutic strategy to ameliorate oxidative stress induced multi-organ dysfunction in sepsis.

## 6. N-Acetylcysteine

NAC is a powerful synthetic antioxidant that scavenges superoxide, H_2_O_2_ and hydroxyl radicals and replenishes endogenous glutathione levels, thereby enhancing the host’s antioxidant defenses [94]. NAC has been shown to suppress the generation of oxygen radicals by human neutrophils and macrophages in vitro [95]; reduce leucocyte-to-endothelial cell adhesion and vascular leakage in rodent endotoxemia [96]; and attenuate the activation of nuclear factor kappa-B (NF-κB) and interleukin-8 levels in sepsis [97]. Such findings provided the impetus for examining the therapeutic potential of NAC to reverse the deleterious effects of oxidative and nitrosative stress in sepsis. 

Multiple experimental studies have investigated NAC either as a prophylactic treatment or as a rescue therapy in early stages of sepsis. In a rodent model of sepsis induced by cecal ligation and puncture (CLP), subcutaneous NAC (20 mg/kg) treatment initiated 3 h after CLP reduced systemic inflammation, oxidative stress and mitochondrial dysfunction, leading to a significant improvement in survival (10% to 40%) [98]. In rodent endotoxemia, intravenous NAC treatment (150 mg/kg bolus + 12.5 mg/kg/h) initiated 20 min after administration of lipopolysaccharide reduced serum creatinine and blood urea nitrogen concentrations, an effect associated with reductions in inflammatory cytokines (TNF-α and IL-6) [99]. However, treatments initiated at such early stages of sepsis are arguably of limited relevance to the clinical setting. This is because patients with sepsis are often diagnosed and then receive interventions in the presence of established multi-organ dysfunction, as determined by sequential organ failure assessment (SOFA) scores [100]. In this regard, intravenous treatment with NAC (150 mg/kg bolus + 20 mg/kg/h) in pigs 12 h after establishment of endotoxemia failed to improve systemic, pulmonary and hepatosplanchnic hemodynamics, or reduce a biomarker of oxidative stress (8-isoprostane), despite elevated glutathione concentrations [101].

Clinical studies that have utilized NAC as an adjunct treatment for sepsis have largely led to disappointing outcomes, particularly when NAC was administered to patients with severe sepsis for longer durations. In most clinical studies, the dose of NAC used in sepsis (150 mg/kg) was based on the regimen in patients treated for acetaminophen poisoning [102]. In patients with sepsis, intravenous NAC administered over a relatively short period (150 mg/kg bolus + 12.5 mg/kg/h for 90 min) significantly increased cardiac index (5.7 (treatment) vs. 5.0 (usual care) L/min/m^2^, *p* < 0.05) and absolute liver blood flow index (3.3 (treatment) vs. 2.7 (usual care) L/min/m^2^, *p* = 0.01). However, NAC administered over a longer period (50 mg/kg over 4 h followed by 100 mg/kg/24 h for 44 h) worsened organ failure (mean SOFA scores at 48 h: 7.7 ± 3.8 (treatment) vs. 5.1 ± 2.1 (usual care), *p* < 0.05) and did not improve microalbuminuria/creatinine ratio in patients with sepsis [103]. Concerningly, administration of NAC in patients diagnosed with septic shock for durations exceeding 24 h caused cardiac depression, characterized by reduced cardiac output and exacerbation of hypotension [104]. Similarly, greater requirements for inotropic support were demonstrated in critically ill patients treated with NAC (150 mg/kg bolus + 12 mg/kg/h) for durations longer than 24 h compared with usual care [105]. The highest known dose of NAC administered to patients with sepsis (3 g every 6 h for 3 days) did not significantly improve patient mortality, but had deleterious effects, including increasing the risk of inflammation in association with worsened AKI [106]. However, there is currently a paucity of pharmacokinetic and pharmacodynamic data regarding NAC in sepsis; and the optimal timing and dosages of NAC with respect to onset, disease progression and severity of sepsis remain unknown. Therefore, further clinical and experimental studies are required before the potential for beneficial effects of NAC in sepsis can be completely discounted.

## 7. Vitamin C

Vitamin C is an essential circulating antioxidant which directly scavenges ROS and RNS [107] and replenishes endogenous glutathione concentrations, thereby regenerating the host’s antioxidant defenses [108]. Similarly to NAC, vitamin C also causes anti-inflammatory actions by inhibiting the activity of NF-κB and the production of pro-inflammatory mediators [109]. However, unlike NAC, vitamin C has pleiotropic properties that may be of further benefit to sepsis. Vitamin C is an immune-stimulant which enhances the activity of macrophages [110] and may have the ability to directly inhibit bacterial replication [111]. In cultured human endothelial cells, vitamin C dose-dependently inhibited the TNF-α-induced expression of intracellular adhesion molecule-1, so it has the potential to reduce microvascular leucocyte plugging and microcirculatory dysfunction [112]. Vitamin C can also reverse BH4 oxidation and elevate BH4-dependent synthesis of nitric oxide from eNOS [113] and/or enhance eNOS bioavailability via a rapid phosphorylation of eNOS-Ser1177 [114], which are effects not observed with antioxidants such as NAC [115]. Vitamin C is also an important cofactor for the synthesis of endogenous vasoconstrictors such as noradrenaline and vasopressin [116], which may further aid in the circulatory management of patients with sepsis who often become unresponsive to vasopressor therapy. Critically-ill patients have abnormally low plasma ascorbate levels [117,118,119], which is compounded by the inability of humans to naturally synthesis vitamin C, providing another reason for the administration of vitamin C during sepsis [120]. Since enteral uptake is insufficient to normalize plasma ascorbate levels due to saturable intestinal sodium-dependent vitamin C transporters (SVCTs), intravenous treatments are required [121].

Experimental studies have provided compelling evidence for the potential benefits of intravenous vitamin C in sepsis. In murine sepsis induced by CLP, pre-treatment with sodium ascorbate (200 mg/kg) prevented vascular leakage by inhibiting induction of iNOS, superoxide production and peroxynitrite formation in skeletal muscle tissue [122]. In the same study, sodium ascorbate reduced CLP-induced superoxide generation by preventing NADPH oxidase activation and uncoupling of eNOS [122]. In mice with fecal-induced peritonitis (FIP), ascorbate treatment at 6 h post-FIP reversed platelet adhesion, improved muscle capillary blood distribution and increased survival, but these beneficial effects were not observed in eNOS knockout mice [123,124]. Pre-treatment with sodium ascorbate (76–200 mg/kg) has been demonstrated to attenuate the reduced pressor responsiveness to vasopressors, including noradrenaline and angiotensin II, observed in CLP mice via inhibition of iNOS activity [125,126]. Such experimental observations have provided the scientific rationale for clinical trials examining the putative benefits of intravenous vitamin C in human sepsis.

Despite the proposed benefits of vitamin C identified in animal models of early sepsis, its efficacy in human sepsis remains controversial. Two small, single-center randomized clinical trials (RCT; *n* = 24 & *n* = 28) showed that intravenous administration of ascorbic acid, at doses ranging from 50 to 200 mg/kg/day, reduced inflammatory biomarkers and SOFA score [127] and improved vasopressor sensitivity (25 mg/kg every 6 h for 3 days) [128]. Another single-center, retrospective before-and-after study (*n* = 47) using a combination therapy of ascorbic acid (1.5 g; 4 times/day) with hydrocortisone and thiamine, identified significant reductions in hospital mortality (8.5% vs. 40.4%, *p* < 0.001), duration of vasopressor use (18.3 ± 9.8 h vs. 54.9 ± 28.4 h, *p* < 0.001) and the requirement for RRT (10% vs. 37%, *p* < 0.05) at 72 h after the intervention [129]. However, subsequent multi-center RCTs, VITAMINS [130], ACTS [131] and ATESS [132], that trialed ascorbic acid at 6 g/day with thiamine with and without corticosteroid for up to 10 days, failed to detect differences in mortality or requirement for RRT in patients with sepsis. The CITRUS-ALI trial which utilized ~16 g/day of intravenous ascorbic acid for 4 days in patients with sepsis, also did not detect improved mean modified SOFA scores, although a reduction in 28-day mortality from 46% to 30% was observed [133]. Sepsis is characterized by a profound systemic inflammatory state, which has been showed to downregulate cellular SVCTs [134,135]. Accordingly, higher doses than already trialed in clinical sepsis may be required to circumvent the decrease in SVCTs and drive vitamin C into cells to confer its intracellular benefits.

Since very high doses of vitamin C have been demonstrated to be safe in patients with burns or cancer [136], the pre-clinical safety and efficacy of a mega-dose of sodium ascorbate (150 g for a 40 kg sheep) was recently investigated in an ovine model of sepsis with established AKI [137]. After 24 h of established sepsis, mega-dose sodium ascorbate treatment effectively reversed renal medullary ischemia and hypoxia in septic sheep, which was accompanied by a complete reversal of AKI, as detected by profound increases in urine flow and creatinine clearance and normalization of plasma creatinine concentration [137]. Sodium ascorbate also significantly reduced the requirements for noradrenaline needed to restore target blood pressure, reduced arterial blood lactate levels and improved lung function in septic sheep [137]. In addition, a critically ill septic patient with COVID-19 induced hypotension and AKI was treated with intravenous sodium ascorbate (60 g), as compassionate use. As in septic sheep, sodium ascorbate reduced plasma creatinine (118 to 84 µmol/L) and increased urine flow (10 to 400 mL/h), and mean arterial pressure was restored despite a complete withdrawal of noradrenaline, at least over the 7 h intervention period [137]. Collectively, these pre-clinical and clinical studies suggest that the optimal dosage, timing and duration of vitamin C therapy are likely to be critical factors that determine its therapeutic efficacy in sepsis, but these factors currently need detailed investigation [120]. Hence, two pilot placebo controlled RCTs are currently underway examining the effects of intravenous mega-dose sodium ascorbate treatment (sodium ascorbate, 60 g and 120 g) on renal outcomes and vasopressor requirements in patients with sepsis (ACTRN12620000651987p; NCT04796636).

## 8. Conclusions

There is evidence that early in sepsis, microcirculatory dysfunction, secondary to inflammation and oxidative stress, results in localized tissue hypoxia and mitochondrial dysfunction, thereby initiating a perpetuating cycle of cellular injury and progressive AKI. Studies of experimental sepsis indicate that the anti-inflammatory and antioxidant actions of NAC and vitamin C confer multi-organ protection, especially when administered either prophylactically or during the early stages of sepsis. However, these putative benefits of NAC and vitamin C seen in animal studies have not always translated to heterogenous populations of patients with sepsis with established multi-organ dysfunction, when administered at various time-points of disease severity over prolonged durations of treatment. Accordingly, preclinical studies in clinically relevant models of sepsis are required to increase our understanding of the mechanisms of action of antioxidants in sepsis and to establish optimal doses given at clinically appropriate time-points of sepsis. Such studies are essential to providing a justified scientific rationale for the design of large, double-blinded, placebo controlled, multi-center RCTs, which are essential to identifying the specific characteristics of patients with septic AKI who would most likely benefit from NAC and vitamin C, with respect to their dosages, timing and durations of treatment.

## Data Availability

The data presented in this review from YRL and CNM laboratories are available on request from the corresponding author.
